# A Microfluidic Single-Cell Cloning (SCC) Device for the Generation of Monoclonal Cells

**DOI:** 10.3390/cells9061482

**Published:** 2020-06-18

**Authors:** Chuan-Feng Yeh, Ching-Hui Lin, Hao-Chen Chang, Chia-Yu Tang, Pei-Tzu Lai, Chia-Hsien Hsu

**Affiliations:** 1 Institute of Biomedical Engineering and Nanomedicine, National Health Research Institutes, Miaoli 35053, Taiwan; 950308@nhri.org.tw (C.-F.Y.); jhlinzzb@tsmc.com (C.-H.L.); vivian@origembiotech.com (H.-C.C.); jethro.taipei@gmail.com (C.-Y.T.); 050871@nhri.org.tw (P.-T.L.); 2 Institute of NanoEngineering and MicroSystems, National Tsing Hua University, Hsinchu 30013, Taiwan; 3 Tissue Engineering and Regenerative Medicine, National Chung Hsing University, Taichung 40227, Taiwan

**Keywords:** microfluidics, single-cell cloning, monoclonal cell lines

## Abstract

Single-cell cloning (SCC) is a critical step in generating monoclonal cell lines, which are widely used as in vitro models and for producing proteins with high reproducibility for research and the production of therapeutic drugs. In monoclonal cell line generation, the development time can be shortened by validating the monoclonality of the cloned cells. However, the validation process currently requires specialized equipment that is not readily available in general biology laboratories. Here, we report a disposable SCC device, in which single cells can be isolated, validated, and expanded to form monoclonal cell colonies using conventional micropipettes and microscopes. The monoclonal cells can be selectively transferred from the SCC chip to conventional culture plates, using a tissue puncher. Using the device, we demonstrated that monoclonal colonies of actin-GFP (green fluorescent protein) plasmid-transfected A549 cells could be formed in the device within nine days and subsequently transferred to wells in plates for further expansion. This approach offers a cost-effective alternative to the use of specialized equipment for monoclonal cell generation.

## 1. Introduction

Monoclonal cells are groups of cells originating from a single parental cell. They have cognate genomic DNA sequences and express similar phenotypes. Monoclonal cells are widely used as cell models to study the function of genes or produce antibodies [1] for use in a wide spectrum of applications, ranging from basic research [2,3] to the production of therapeutic drugs [4,5,6].

To construct monoclonal cell lines, the genomic DNA of cells must be modified by transfecting foreign DNA into the cells. However, transfected cells usually have highly diverse gene complements, due to the random insertion of target genes and the process of gene amplification [7]. Highly expressing cells are rare in these heterogeneous cell populations [8,9,10]. Transfected cells also have different cell viabilities, due to cell damage from the transfection process. As a result, screening and selecting for large numbers of monoclonal cells with a desired phenotype and high reproductive capacity are necessary [1,11,12,13]. Therefore reducing the time of the screening and selection processes could have significant financial implications for protein productions in which a right production clone (i.e., clone that can synthesize the required protein at high productivity) usually takes months to generate [14].

Current single-cell cloning (SCC) techniques can be categorized as dilution cloning or deterministic. Dilution cloning methods are based on the use of increasing dilutions of a cell culture to produce low-density parental cell suspension aliquots (between 0.25 and 1 cells/culture), which are plated to produce single-cell-derived colonies. The cell dilution procedure can be performed using micropipettes and 96-well plates, which are readily available in general laboratories [15,16,17,18]. Alternatively, fluorescence-activated cell sorting (FACS) may be used to assign cells into plate wells to reduce the amount of labor required. Other manual methods, such as cloning-ring methods [17,19] or using agarose-based medium [19], also use low-density parental cell populations to selectively collect single-cell-derived colonies. These dilution-based methods often require multiple rounds of SCC to increase the probability of monoclonality, because single cells are difficult to observe in the wells of commercial plates [18,20]. Since one round of the cell cloning process can take about a month [1], generating monoclonal cells using dilution-based methods is time-consuming.

To eliminate repetitive cloning, several deterministic methods have been developed using single-cell images to assure monoclonality [21]. Pai et al. reported a microtable array device into which a low–cell-density suspension can be loaded to allow cells to attach and grow on arrayed tables. One-cell-per-table events can be observed with a microscope to validate the monoclonality of the growing cells. However, to release and transfer the expanded cells from the device, specialized laser equipment is required [22]. Matsumura et al. reported a microculture chamber device in which improved culture conditions can be provided for growing cell colonies from difficult-to-culture cells, such as induced pluripotent stem cells. However, validating the monoclonality requires the use of a time-lapse microscopy image system [23]. In recent years, equipment involving integrated robots and imaging systems has been developed to validate monoclonality for cell line generation [21,24]. However, this equipment is not readily available for general users.

In this paper, we report the development and use of a disposable microfluidic chip device with which single cells can be isolated and grown into monoclonal colonies, which can subsequently be transferred from the device to the wells of conventional plates for further expansion. The device can be used in general laboratories, because it uses conventional micropipettes, tissue punchers, and microscopes.

We used actin-GFP plasmid-transfected A549 cells to demonstrate the usability of the device and showed that the transfected cells were successfully isolated, expanded into colonies, and subsequently transferred to wells in the plates, to form A549 cell clones with actin-GFP expression levels that were higher than those of the transfected pool of heterogeneous cells.

## 2. Materials and Methods

### 2.1. Device Design and Fabrication

The SCC microfluidic devices were modified and fabricated in accordance with our previous publication [25]. SU-8 (MicroChem, Newton, MA, USA) was patterned on silicon wafers to create masters through photolithography. The masters were then used as molds, on which a Sylgard 184 (Dow Corning, Midland, MI, USA) PDMS prepolymer mixed with its cross-linker at a 10:1 ratio was poured and allowed to cure in a conventional oven at 65 °C for three hours. A puncher with a 0.75 mm inner diameter (WellTech, World Precision Instruments, Sarasota, FL, USA) was used to punch inlet holes for the fluidic channel of the PDMS device. After brief oxygen plasma treatment, the PDMS replicas were aligned, brought into contact, and placed in an oven at 65 °C for 24 h to achieve permanent bonding between the PDMS replicas.

### 2.2. Cell Culture and Maintenance

A549 cells (BCRC, Hsinchu, Taiwan) were used as model cells in this study. The A549 cells were cultured in DMEM basal medium (Gibco, Co Dublin, Ireland) with 10% fetal bovine serum (FBS, HyClone, Boston, MA, USA) and 1% antibiotic (penicillin/streptomycin, Gibco). The cell cultures were passaged using the recombinant reagent Accumax™ (Innovative Cell Technologies, San Diego, CA, USA) following the manufacturer’s standard protocol at 70–80% confluence.

### 2.3. SCC Device Preparation for Single-Cell Capture

Prior to experiments, the SCC devices were filled with deionized water and soaked in a deionized water-filled container in a desiccator, to remove air bubbles in the microchannel. Subsequently, the degassed SCC devices were exposed to UV light to sterilize for 30 min. To prevent immediate cell adhesion to the PDMS surface, 5% bovine serum albumin (Bersing Technology, Hsinchu, Taiwan) in 1 × PBS was injected into the microfluidic channel and incubated at 37 °C for 30 min.

### 2.4. Single-Cell Capture and Cloning in SCC Device

For single-cell capture experiments, the optimization parameters used were as presented in our previous publication [25]. Briefly, A549 cells were resuspended at a concentration of 1.5 × 10^6^ cells per mL using Accumax™. Cells were loaded into the SCC device by inserting the 200 μL tip into the device’s inlet hole to manually inject the cells. This step quickly loads the cells into the microchannel and covers the area of capture wells. The device was allowed to stand for five minutes. During this time, some cells in the microchannel descended down the capture wells by gravitational force. Subsequently, a syringe run by a syringe pump (Harvard Apparatus, Harvard Bioscience, Holliston, MA, USA) was connected to the inlet of the SCC device via Teflon tubing (polytetrafluoroethylene; inner diameter, 0.51 mm; outer diameter, 0.82 mm; Ever Sharp Technology, Inc., Hsinchu, Taiwan) to load 400 μL of the cell culture medium into the device at 600 μL min^−1^, washing out non-captured cells. Finally, the inlet and outlet holes were sealed with plugs, and the device was flipped upside down to transfer the captured cells into the culture wells by gravitational force. The device was then placed in a standard cell culture incubator at 37 °C with 5% CO_2_, and the medium was changed every two days for seven to nine days.

### 2.5. Characterization of Single-Cell Events by Limiting Dilution, SCC Device, and FACS

Cells cultured on 10 mm plates at 80% confluence were pre-stained with 10 µM calcium AM (acetoxymethyl) fluorescence dye (L3224, Thermo Fisher, Waltham, MA, USA), incubated for 20 min at room temperature, and protected from light for easy identification of the cells in plates or devices. For limiting dilution, cells were detached using Accumax^TM^ and resuspended into 96-well plates at 0.3 cells per 100 µL using an eight-channel pipette. Sixty wells were loaded per 96-well plates. Images were obtained by scanning the whole plates 30 min after cell loading. Single-cell events were validated and quantified using scanned images of the whole plates.

For FACS, cells were detached using Accumax^TM^ and resuspended at 1 × 10^6^ cells/mL in PBS. To each well of a 96-well culture plate, 100 µL DMEM basal medium (Gibco, Gaithersburg, MD, USA) with cell culture medium was pre-added, and each cell was sorted into 1 of 60 wells of the culture plate using a fluorescence-activated cell sorter (FACSAria, BD Biosciences, San Jose, CA, USA). Then, 100 μL PBS was added to the side wells of the plates to prevent evaporation. Images were obtained by scanning the whole plates 30 min after cell sorting, to validate and quantify single-cell events. For the SCC device, a suspension of 1.5 × 10^6^ cells/mL was prepared for loading. The protocol followed was as previously discussed for single-cell capture and cloning by the SCC device. After flipping the device, scanned images of culture-well arrays of the SCC device were obtained after 10 min. Single-cell events were validated and quantified using scanned images of the whole device.

### 2.6. Transferring and Releasing Cell Colonies from the SCC Device

In a biosafety cabinet, the plasma bonded side of the PDMS was sliced off using a knife. The culture wells that had previously been selected were punched out using a 0.75 mm tissue puncher (WellTech, World Precision Instruments), producing PDMS plugs. The plugs were collected individually and transferred into 96-well culture plates containing Accumax^TM^ for further cell proliferation. After transferring the PDMS plugs into individual wells, the plates were incubated at 25 °C for five minutes and then placed on a microplate vortex device (MicroPlate Genie, Scientific, Inc., Ocala, FL, USA) for one minute at 10 rpm to completely release the cells. Fluorescence images were obtained by scanning the plates immediately. The cells in the cell images were counted and quantified using the imaging software NIS elements AR (Nikon, Japan). Finally, 100 µL DMEM medium was added, and the plate was placed in a 37 °C incubator with 5% CO_2_.

### 2.7. Quantitation of Cell Transfer and Release from the PDMS Surface of a Device to a 96-Well Plate

A549 cells were manually loaded into the device using a 200 µL pipette tip at cell densities of 1 × 10^5^ cells/mL (low density), 2 × 10^5^ cells/mL (medium density), or 4 × 10^5^ cells/mL (high density). After 24 h of culture, cells were stained with a 20 µg/mL membrane dye (DiI(C12)3, BD Biosciences, USA) for 20 min. The device was opened, and the culture wells were punched out at random using a tissue puncher. The cells were then released to 96-well culture plates using the transfer and release protocol described previously. Fluorescent images were obtained by scanning the device and plate. The cells were counted before punching out the wells from the device and after releasing to 96-well plates, using NIS elements AR imaging software, to quantify the efficiency of cell transfer from the device to the 96-well plate. The cells were counted before and after punching out the wells from the SCC device, to verify whether there was contamination of cells from neighboring wells during the transfer process.

### 2.8. Single-Cell Growth 

After isolating single cells, images were obtained within 10 min by scanning the whole SCC devices. The locations of the wells that captured single cells were recorded. Images at six time points were obtained from day 0 to day 8. The wells that originally had a single cell at day 0 were monitored for cell growth. Cell division was counted at days 1, 2, 4, 6, and 8 in scanned images, using NIS elements AR.

### 2.9. Single-Cell Growth after Release from PDMS Plugs 

Twenty-four hours after cell releasing, the cells were stained with 10 µM of calcium AM fluorescence dye (L3224, Thermo Fisher) at days 1, 3, and 6, and fluorescence images were obtained. The cells were counted and quantified using NIS elements AR software.

### 2.10. LifeAct Plasmid Transfection 

A549 cells were cultured in six-well plates in DMEM medium supplemented with 10% FBS and grown to 80% confluency. The p^CMV^LifeAct-Tag^GFP2^ plasmid (Ibidi, USA) was transfected with 2.5 µg by simultaneous use of 7.5 µL of transfection reagent (TransIT-LT1, Mirus, Madison, WI, USA), according to the manufacturer’s instructions. The medium was changed after 24 h, and the cells were maintained with 1 mg/mL G418 (A1720, Sigma, USA) every two days for two weeks.

### 2.11. Clonality-Validated A549 LifeAct Cell Line Established by the SCC Device

LifeAct A549 stable cell pools were loaded into the SCC device as previously described. Images of the whole devices were acquired at days 0, 1, 3, and 9. Single-cell-derived colonies were identified by GFP expression in time-lapse images and were then transferred and released to 96-well plates for continuous cell expansion.

### 2.12. A549 LifeAct Monoclonal Cell Line Characterization

The GFP expression from five individual monoclonal LifeAct A549 cell lines was quantified using flow cytometry (FACS Calibur, BD bioscience, USA) with the Cell Quest Pro software (Becton Dickinson). LifeAct monoclonal cells were stained with 100 nM rhodamine phalloidin (Cytoskeleton, Inc., Denver, CO, USA) to confirm actin localization using confocal microscopy (Leica TCS SP5 II, Germany).

### 2.13. Acquisition of Cell Images

All images were obtained using an inverted fluorescence microscope (TiE, Nikon, Japan) with a cooled CCD camera (Retiga 4000DC, QImaging, Canada) and NIS elements AR software.

### 2.14. Statistical Analyses

All experiments were performed in triplicate, and data are presented as mean ± standard deviation. One-way analysis of variance and Student’s *t*-tests were used for the comparison of groups.

## 3. Results and Discussion 

### 3.1. Design and Operation of the SCC Device

**Figure 1:**
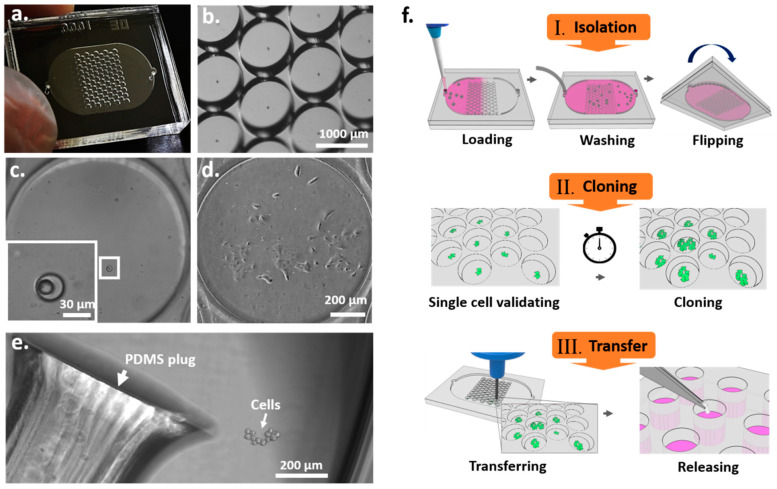
Microfluidic device for single-cell cloning (SCC). (**a**) Image of the pair-wells device for single-cell trapping and cloning. (**b**) Image of the clone wells and trap wells of the device taken under a dissecting microscope. (**c**) The inset image shows an enlargement of a trap well within a single cell. (**d**) A single-cell-derived clone proliferated in the clone well after nine days. (**e**) The cell colony was then released from the SCC device by punching the PDMS surface. The PDMS plug was collected and transferred to a 96-well plate for further cell proliferation. (**f**) Schematic diagram of the principal operation of the SCC device. There are three significant steps for establishing monoclonal cell lines: single-cell isolation, cloning, and transfer.

**Figure 2:**
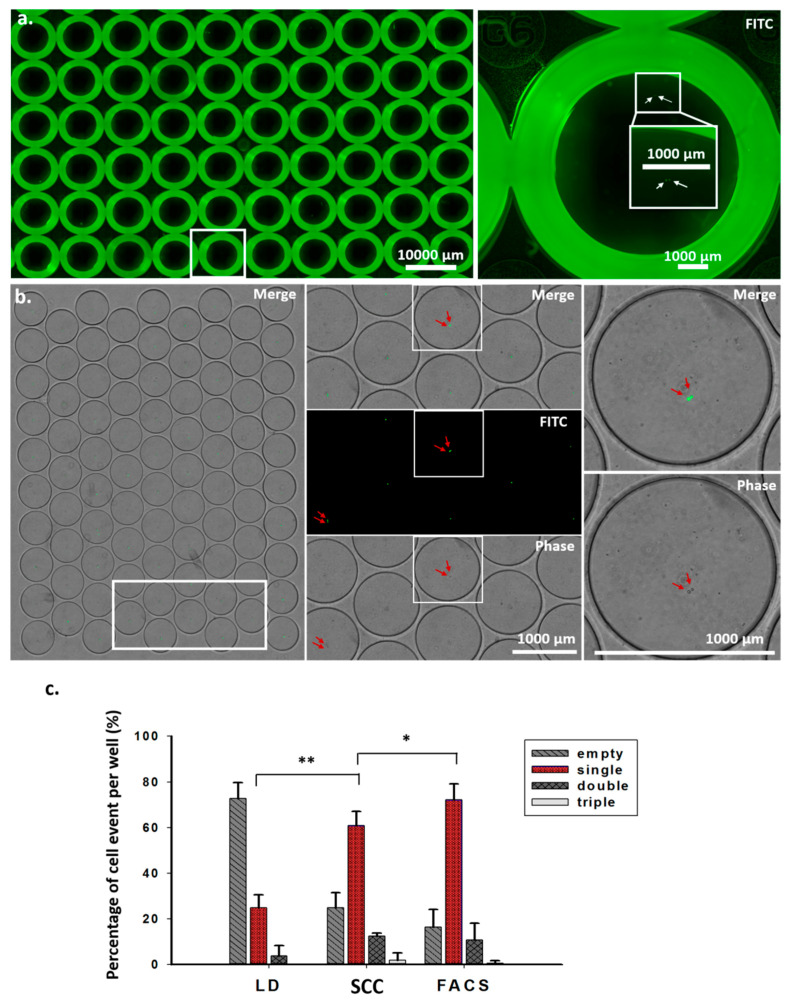
Validation and quantitative analysis of cell events in wells from scanned images. (**a**) Images were obtained by scanning 96-well plates after the individual cells were sorted through FACS for 30 min. Enlarged images show double cells in a well (right). (**b**) Images were obtained by scanning a microfluidic device within 10 min after the single cell was sorted (left). The white frame from the left image was enlarged (middle), showing merge, fluorescence, and phase images. Two arrows in each well indicate double cells. Enlarged images show merge and phase images of double cells (right). (**c**) Quantitative analysis of cell events per well after single-cell sorting using the limiting dilution method, the SCC device, and FACS. * *p* < 0.05., ** *p* < 0.005. Student’s *t*-test. *n* = 4, two independent experiments.

To improve the efficiency of SCC, we designed a chip consisting of 86-unit pairs of wells for single-cell trapping and cloning. The microchannel of the device had an area of 12.75 × 20.25 mm^2^, a height of 100 µm, and a total chamber volume of 40 µL (Figure 1a,b). The trap wells were 32 ± 4.4 µm in diameter and 34.7 ± 1.93 µm in depth (Supplementary Figure S1e) and were designed to isolate single cells, resulting in a significant increase in cell trapping efficiency compared to the Poisson distribution (Figure 2c and Table 1). High-resolution images of the microscale device show single cells in the trap wells (Figure 1c), thus eliminating concerns about cell clustering that occur in the dilution methods. The clone wells, which were 1019 ± 14.46 µm in diameter and 392 ± 7.66 µm in depth (Supplementary Figure S1f), could each provide an area adequate for a single cell to grow into a colony, a process which took about seven to nine days (Supplementary Movie S4). The diameter of the clone wells was designed to be visually distinguishable to allow for straightforward transfer of colonies without microscopic observation, since the locations of colonies in the device can be determined by acquiring images before cell transfer. As shown in Figure 1d, a single-cell-derived colony was formed in a clone well on day 8 and transferred from the device to a 96-well plate for subsequent expansion (Figure 1e). The throughput of the device could be increased by increasing the footprint of the device to contain more units of the well pairs.

The operation of the SCC device involves several steps. (1) Single-cell isolation: a cell suspension is loaded into the device and allowed to stand for two minutes to let the cells fall into the trap wells by gravity (Supplementary Figure S2). Non-trapped cells are washed out before sealing the inlet holes (Supplementary Figure S2 and Supplementary Movie S1). Subsequently, the device was flipped to allow the captured cells to fall from the trap wells into the clone wells by gravity (Supplementary Figure S2 and Supplementary Movie S2). (2) Single-cell validation and cloning: images of the entire SCC device can be taken after 10 min. The number of cells was identified for each clone well, and single-cell capture efficiency was evaluated (Figure 2b,c). Images taken after cell loading and at different time points during cell culture can be used to reveal the presence of a single cell and its growth, to confirm the monoclonality of the cells in the wells. Trap wells that contain only one cell are identified, and their positions are recorded. Afterward, images of the recorded wells are taken at different time points to evaluate the population number and growth rate of the single-cell-derived colonies. (3) Colony transfer and expansion: a 96-well plate is prepared beforehand by adding 50 µL of Accumax^TM^ cell dissociation solution into each well. The PDMS device is cut open to expose the clone wells. Clone wells that have been previously observed to display sufficient cell growth are manually punched out using a tissue puncher. Each cell-containing PDMS plug is then transferred into a well on a 96-well plate. Once the cells are released from the PDMS plug, they continue to grow into a larger cell population (Figure 1e). The SCC chip-based approach can increase the efficiency of monoclonal cell generation by increasing single-cell events with a special microchannel design, allowing straightforward validation of monoclonality and transfer of cells, while using equipment accessible for general laboratories.

### 3.2. The SCC Device Offers High-Efficiency Single-Cell Isolation and Identification

For monoclonal cell generation, validating single-cell events is required but is very difficult, if not impossible, to perform using a conventional well plate. As shown in Figure 2a, fluorescence labeling is required to visually identify cells in a 96-well culture plate. A strong background fluorescence near the edges of the wells can interfere with cell identification. For this reason, the use of several cycles of re-cloning has become a standard procedure for dilution-based methods for the generation of monoclonal cells. In our miniaturized device, due to the small size of a clone well, which is around 100 times smaller than that of a standard 96-well plate, identifying single cells has become straightforward. The small footprint of the device means that less time is required to scan or image the cells (Figure 2b). We compared the single-cell efficiencies of limiting dilution, SCC, and FACS methods. As shown in Table 1 and Figure 2c, the single-cell efficiency of the SCC device was 60.98%, significantly higher than that of the conventional limiting dilution method, at 24.98%, and slightly lower than that of the FACS-assisted method.

### 3.3. Single Cells Can Proliferate and Maintain Monoclonality in Clone Wells

**Figure 3:**
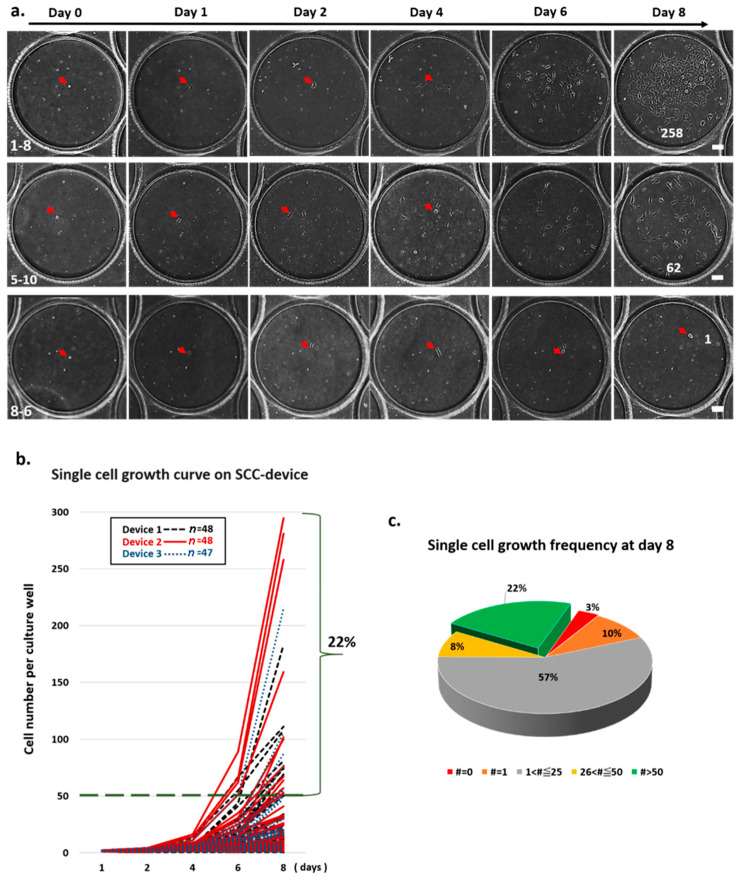
Validation of single cell’s morphology and cell growth in an SCC device. (**a**) Time-course images from the clone well show cell growth on the SCC device over time. (**b**) Single-cell division in each well was monitored and counted using scanned images from day 0 to 8. Of the single-cell-derived colonies, 22% proliferated to more than 50 cells and formed a cluster. (**c**) Frequency of cell growth on day 8 is represented as a pie graph. Each color represents the number (#) of cells counted on day 8.

We tracked the division curve of every single cell in the clone wells from three individual SCC devices after cell isolation (Figure 3a). Of the 86 wells of a SCC device, 48 were found to contain a single cell in each well in two SCC devices, while 47 wells were found to contain a single cell in each well in one SCC device. As shown in Figure 3b, heterogeneous cell growth rates were observed. Of the tracked wells, 22% were found to contain more than 50 cells after eight days of cell culture (Figure 3b,c). We also observed that a single cell may not have grown or may have died from apoptosis (Supplementary Movie S5) during cell culture. Using time-lapse microscopy, we showed that each clone well was able to contain the cells inside the well to prevent cell cross contamination during SCC (Supplementary Movie S4).

### 3.4. Monoclonal Colonies Can Be Transferred and Retain Their Purity and Viability

**Figure 4:**
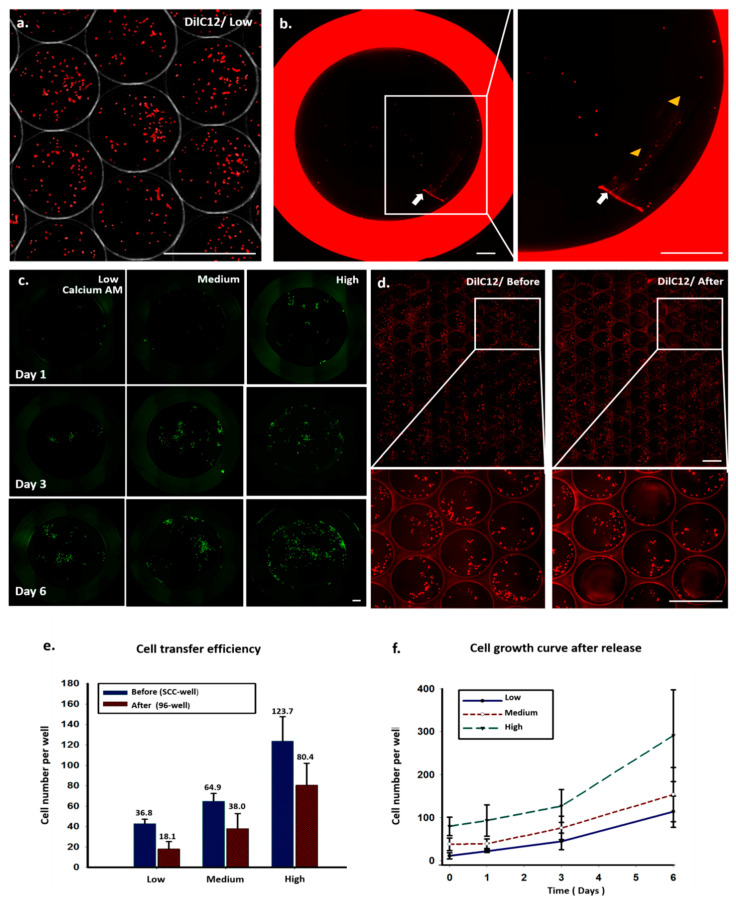
Quantitative analysis of cell transfer efficiency and cell growth curve by scanned images. (**a**) A549 cells were pre-stained with DilC12 dye and cultured in an SCC device for 24 h. (**b**) Image of a 96-well plate after transferring the PDMS plug (white arrow) from the SCC device. Arrowheads indicate cells being released from the PDMS plug after constant shaking. (**c**) Time-course images of cell growth of released cells from day 1 to 6. (**d**) Images of SCC device before and after punching out the wells. Enlarged images show the cells’ distribution, and localization in neighboring wells did not change after punching. (**e**) Cell transfer efficiency and (**f**) cell growth curve after release at different initial cell seeding densities.

After eight days of culture, the colonies with cell numbers greater than 50 were considered to be suitable for transfer from the SCC device to 96-well plates. To quantify cell transfer efficiency, as measured by the number of cells transferred per number of cells in a clone well, we seeded A549 cells of low, medium, and high concentrations into SCC devices; stained the cells with fluorescent DilC12 dye after 24 h of cell culture; and counted the numbers of cells in the clone wells (Figure 4a and Supplementary Figure S4). Some of the clone wells were subsequently punched to transfer cells to a 96-well plate, and the numbers of the cells in the wells of the 96-well plate were counted to calculate the cell transfer efficiency (Figure 4b and Supplementary Figure S4). Some cells were lost during the transfer and release steps because of the smaller punch site; the tissue puncher’s diameter was 0.75 mm, which is smaller than that of the clone well. Others were lost because some cells were not completely released from the PDMS plug (Figure 4e). We found that the cell transfer efficiency could be increased by increasing the cell number in the clone well (Figure 4e). This correlation may be due to the increased numbers of cells congregating in the center of the clone well in a large cell population, whereas a small population of cells tended to have more cells located at the perimeter of the clone well. To minimize the probability of cell cross contamination in the clone wells during the PDMS punching, after each punching, the device was washed with fresh medium. We used images of the clone wells taken before and after punching several wells to examine whether the cell distribution was changed (Figure 4d). The enlarged images show that the numbers of cells in the surrounding clone wells were not changed, indicating that these wells were not contaminated with other cells.

The cells in the 96-well plate were stained with calcium AM fluorescence dye 24 h after cell transfer, to measure cell viability and cell numbers at different time points (Figure 4c,f). Most of the transferred cells were viable and continued to proliferate at good growth rates, indicating that the cell transfer procedure did not cause noticeable cell damage.

### 3.5. Application of the SSC Device for Generating Monoclonal Genetically Modified Cells 

**Figure 5:**
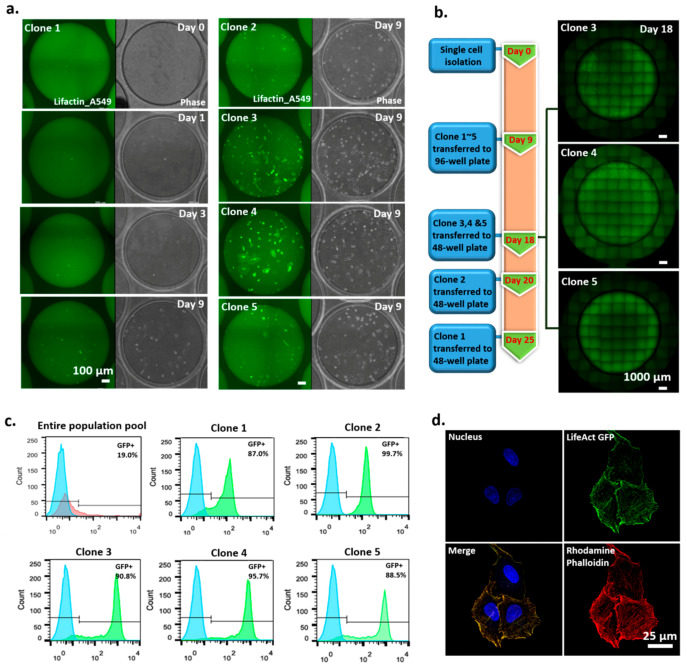
Clonality-validated monoclonal cell lines established by an SCC device. (**a**) Time-course images of single-cell-derived clone 1 (left) and images of single-cell-derived clones 2–5 at 9 days (right). (**b**) Timeline of how clonality-validated monoclonal cell lines are obtained. (**c**) Analysis of GFP expression level of monoclonal cell lines by FACS. (**d**) Verified colocalization of LifeAct-GFP and rhodamine phalloidin.

We used actin–GFP-transfected non-small cell lung cancer A549 cells as a model to demonstrate the applicability of the SCC device for monoclonal cell generation. After nine days of culture, actin–GFP-transfected A549 cell colonies formed in the SCC device and were transferred to a 96-well culture plate (Figure 5a). Subsequently, some of the colonies could be expanded into larger populations and transferred to 48-well plates at different time points, depending on their proliferation rates (Figure 5b). Our demonstration showed that, using the SCC device, three single-cell-derived colonies (clones 3–5) of actin–GFP-transfected A549 cells could be obtained in 18 days.

We measured the GFP expression levels of the five colonies to compare their GFP expression levels with those of the entire population. Our results (Figure 5c) show that, compared to the entire population pool, the five colonies expressed higher, more distinct, and narrower-banded GFP signals, indicating the monoclonality of the colonies. Of the five clones, clones 3 and 4 had high proliferation rates and high protein expression. We then used rhodamine phalloidin, an established fluorescent dye that stains actin protein [26,27], to identify the location of actin fibers in the cells of the clones. We found that the GFP signal in the cells was colocated with the rhodamine phalloidin staining signal (Figure 5d), indicating the successful integration of the actin GFP gene into the transfected cells.

## 4. Conclusions 

We described a new microfluidic chip-based method by which the generation of monoclonality-validated cell lines can be performed with a simple syringe pump and regular pipettes. This approach offers a cost-effective alternative to the use of specialized equipment for monoclonal cell generation shown in Table 2. The usability of the device was demonstrated by showing that clonal generation and selection of actin–GFP-transfected A549 cells could be achieved within 18 days. This method can also be utilized for cloning other cell types since the dimensions of the trap wells may be adjusted for optimal single-cell capture efficiency for the cells of interest [25].

## Figures and Tables

**Table 1:** Comparison of cell events per well after single-cell isolation by limiting dilution, single-cell cloning (SCC) device, and fluorescence-activated cell sorting (FACS). In limiting dilution, 0.3 cells/aliquot were seeded into 96-well plates. The SCC device has a higher single-cell capture efficiency than limiting dilution. Although lower than that of FACS, it is still an advanced method for single cell per well event validation.

Limiting Dilution		SCC Device		FACS	
(0.3/Cells/Aliquot) 96 Well Plate		Clone Well		96 Well Plate	
Cell Events/Well	Percentage	Cell Events/Well	Percentage	Cell Events/Well	Percentage
0	72.27%	0	24.81%	0	16.35%
1	24.98%	1	60.86%	1	72.18%
2	3.88%	2	12.41%	2	10.8%
3	0	3	1.9%	3	0.55%

**Table 2:** Comparison of SCC device, limiting dilution, and image cell sorter.

	SCC Device	Limiting Dillution	Image Cell Sorter
Time-Consuming	No (One Run)	Yes (Multiple Runs)	No (One Run)
**Labor-Intensity**	Low	High	Low
**Costly Equipment**	Low	Low	High
**Reagent Consumption**	Low (Microscale)	High	High

## Supplementary Materials

The following can be found at https://www.mdpi.com/2073-4409/9/6/1482/s1, Figure S1: Quantitative measurement of trap wells/clone wells’ master and PDMS; Figure S2: Three steps for single-cell isolation by an SCC device; Figure S3: Single-cell sorting by FACS; Figure S4: Cell transfer efficiency; Movie S1: Washing out non-trapped cells. After loading the cell suspension in an SCC device, the cells were allowed to stand for two minutes before recording to allow cells to fall down the trap well by gravity. Non-trapped cells were then washed out by loading 600 µL medium to the device at a flow rate of 400 µL/min. The movie shows non-trapped cells being washed out while captured cells remain in the trap wells; Movie S2: Single cell falling down the trap well after flipping the device. The recording shows a single cell falling slowly by gravity from the trap well to the clone well after flipping the device; Movie S3: Single cells could be cloned in the device. The movie shows a time-lapse recording of single-cell division and proliferation from day 0 to 3; Movie S4: Cells are cloned without contact from neighboring wells. Cells in each well grow independently from each other. Some single cells have faster growth rates, while others have slower rates. The cells’ growth is restricted by the clone well, so contamination from contact with neighboring wells is avoided; Movie S5: Some single cells die during cell culture. The movie shows a cell dying 16 h after single-cell isolation.
