# Supplementary material for: A Microfluidic Single-Cell Cloning (SCC) Device for the Generation of Monoclonal Cells
Source: Cells. 2020 Jun 18;9(6):1482. doi: 10.3390/cells9061482 (PMC7349811; doi:10.3390/cells9061482)
Supplement: Supplementary file 1 [file cells-09-01482-s001.zip › cells-805708.Suppl.Final.docx]

**Supplementary Information**


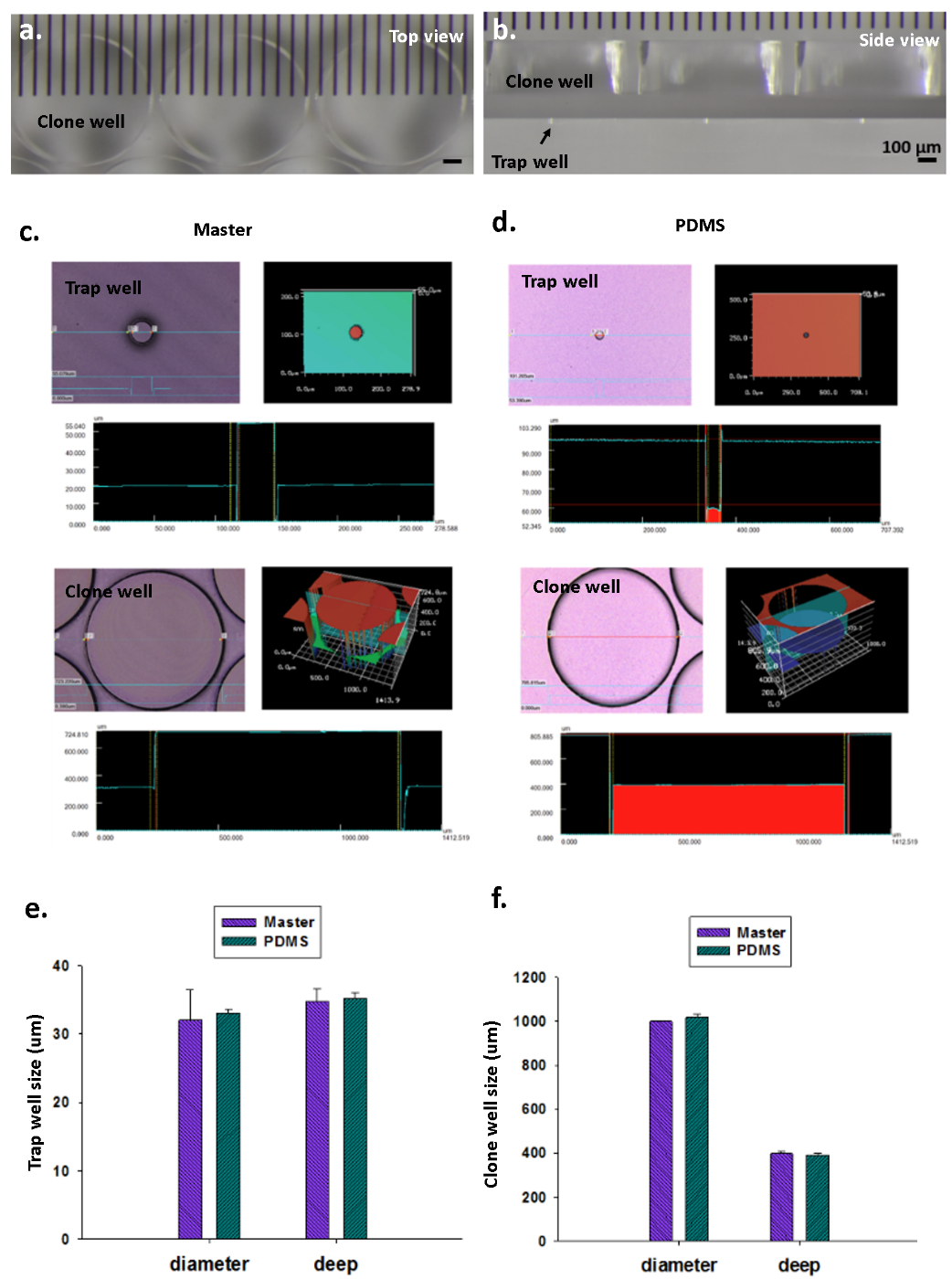


**Supplementary Figure S1. Quantitative measurement of** **trap wells/clone wells’ master and PDMS.** (**a**) Top view of the device. (**b**) Cross-sectional view of the device. (**c**) Master of trap wells/clone wells as measured by 3D profile measurement (VK-X100, KEYENCE, Japan). (**d**) PDMS of trap wells/clone wells were measured by 3D profile measurement (VK-X100, KEYENCE, Japan). (**e**) Quantitative trap well size. n=6. (**f**) Quantitative clone well size. n=6.


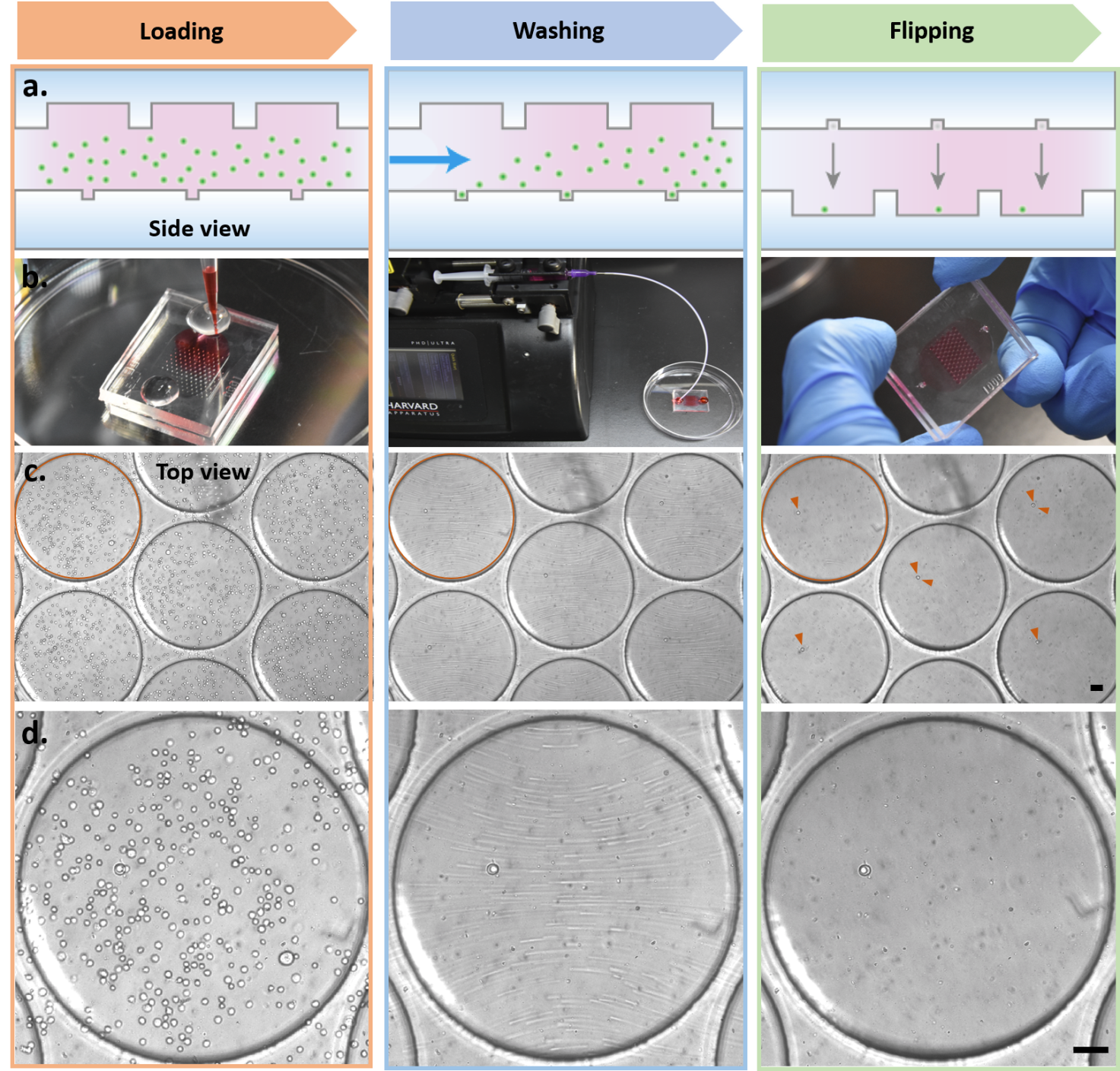


**Supplementary Figure S2. Three steps for single-cell isolation by an SCC device. (a)** Side view of the SCC device during single-cell isolation. (**b**) Photos show the actual operation of the device. (**c**) Images under 4x phase microscopy. (**d**) Enlarged image of one of the clone wells to show details of the capture of a single cell in the trap well.


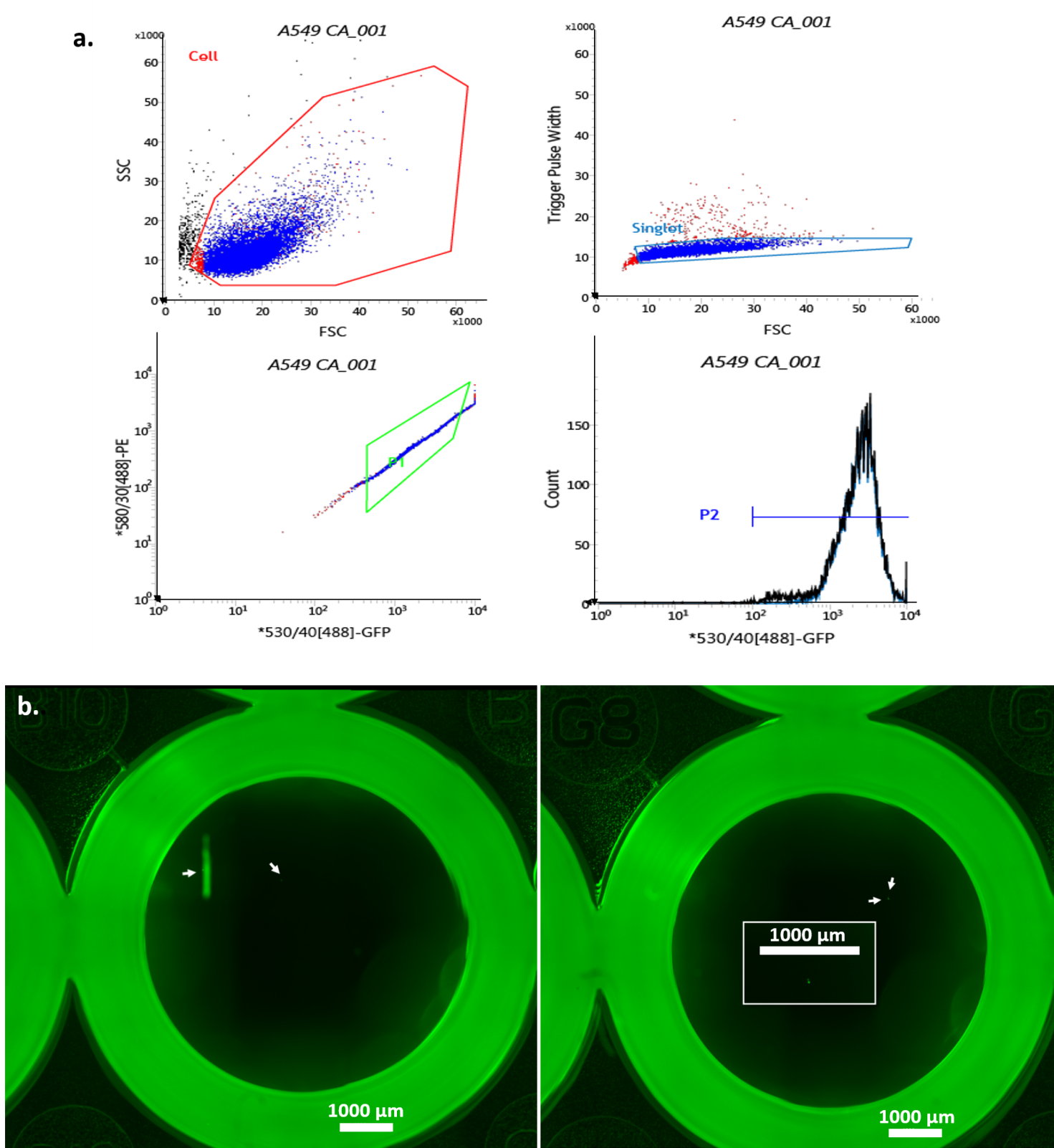


**Supplementary Figure S3. Single-cell sorting by FACS.** (**a**) A549 cells were pre-stained with calcium AM for single-cell sorting and cell events observed using FACS. FACS dot plots and histogram plot show the condition of cells during single-cell sorting. (**b**) Images of 96-well plate after single-cell sorting by FACS. After sorting, 12 wells were found to have double cells in 96-well plate. The arrow shows cells stained with calcium AM. Validating single cells in multi-wells, even with fluorescence, is a challenge. Scale bar, 1000 µm.


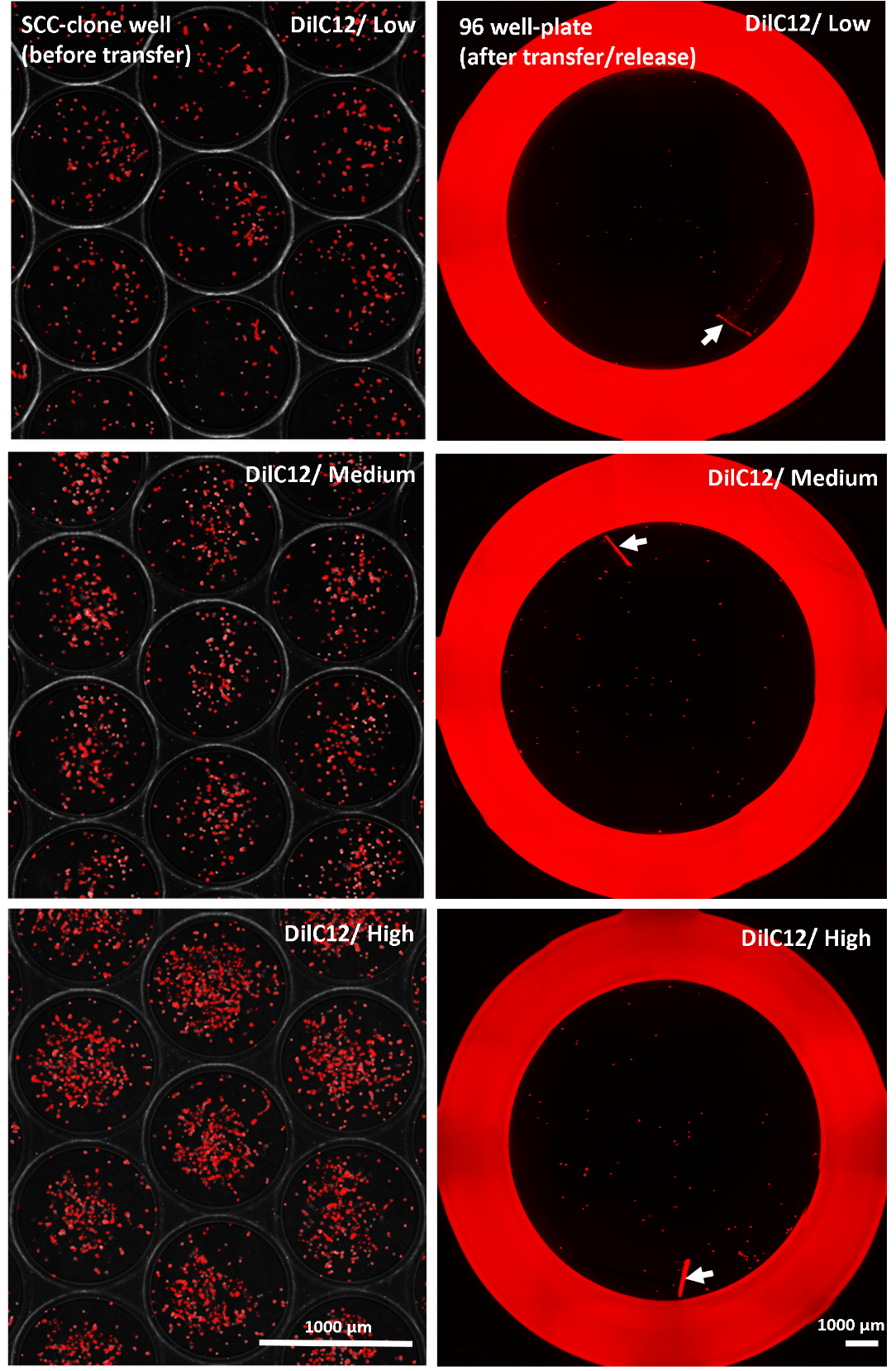


**Supplementary Figure S4. Cell transfer efficiency**. Images of DilC12-stained A549 cells cultured in an SCC device and released cells in 96-well plates. Left panels show different initial seeding densities of A549 cells in SCC clone wells. Right panels show released cells in 96-well plate from PDMS plugs (white arrow).

**Supplementary Movies:**

**Movie S1: Washing out non-trapped cells.** After loading the cell suspension in an SCC device, the cells were allowed to stand for two minutes before recording to allow cells to fall down the trap well by gravity. Non-trapped cells were then washed out by loading 600 uL medium to the device at a flow rate of 400 uL/min. The movie shows non-trapped cells being washed out while captured cells remain in the trap wells.

**Movie S2: Single cell falling down the trap well after flipping the device.** The recording shows a single cell falling slowly by gravity from the trap well to the clone well after flipping the device.

**Movie S3. Single cells could be cloned in the device.** The movie shows a time-lapse recording of single-cell division and proliferation from day 0 to 3.

**Movie S4. Cells are cloned without contact from neighboring wells.** Cells in each well grow independently from each other. Some single cells have faster growth rates, while others have slower rates. The cells’ growth is restricted by the clone well, so contamination from contact with neighboring wells is avoided.

**Movie S5. Some single cells die during cell culture.** The movie shows a cell dying 16 hours after single-cell isolation.
